# Assessing onset, prevalence and survival in mice using a frailty phenotype

**DOI:** 10.18632/aging.101692

**Published:** 2018-12-18

**Authors:** Cory W. Baumann, Dongmin Kwak, LaDora V. Thompson

**Affiliations:** 1 Divisions of Rehabilitation Science and Physical Therapy, Department of Rehabilitation Medicine, Medical School, University of Minnesota, Minneapolis, MN 55455, USA; 2 Department of Physical Therapy and Athletic Training, Boston University, Boston, MA 02215, USA; * Equal contribution

**Keywords:** aging, disease, function, muscle, obesity, physiology

## Abstract

Little is known whether frailty assessments in mice are capable of distinguishing important characteristics of the frailty syndrome. The goals of this study were to identify the onset and the prevalence of frailty across the lifespan and to determine if a frailty phenotype predicts mortality. Body weight, walking speed, strength, endurance and physical activity were assessed in male C57BL/6 mice every three months starting at 14 months of age. Mice that fell in the bottom 20% for walking speed, strength, endurance and physical activity, and in the top 20% for body weight were considered to have a positive frailty marker. The onset of frailty occurred at 17 months, and represented only 3.5% of the mouse cohort. The percentage of frail mice increased with age until basically every mouse was identified as frail. Frail, pre-frail, and non-frail mice had mean survival ages of 27, 29 and 34 months, respectively. In closing, frail mice lack resilience; in that, multiple tissue/organ systems may deteriorate at an accelerated rate and ultimately lead to early mortality when compared to non-frail mice. Identifying the onset and prevalence of frailty, in addition to predicting mortality, has potential to yield information about several aging processes.

## Introduction

Frailty is an age-associated biological syndrome characterized by an increased vulnerability to adverse global health outcomes, a reduced capacity to react to stressors and an overall loss in physiological function [1,2]. Consequently, frail individuals are at a greater risk of falls, dependency, disability, institutionalization, hospitalization and mortality [3,4]. Two of the most commonly used clinical frailty assessments are the phenotype model and the frailty index. The frailty phenotype, proposed by Fried et al. [4], focuses on five specific criteria for unsuccessful physiological aging: unintentional weight loss, weakness, poor endurance/exhaustion, slowness, and low activity. On the other hand, the frailty index developed by Rockwood and colleagues [5,6] conceptualizes frailty using a more multidimensional approach, and is based on a comprehensive geriatric assessment that measures deficits in physiological, psychological, cognitive and social function. Using these approaches approximately 5-10% of community-dwelling individuals 65 years or older are considered frail with the prevalence of frailty increasing with age [1,7,8], highlighting the importance of frailty research.

To date, unanswered questions in the field of frailty are largely due to the inherent limitations of clinical studies, including the ethical, logistical, and biological complications of working with humans, in particular older individuals [9]. With the recent development of mouse models of frailty, reverse translated from the human frailty models, some of these unanswered questions are currently being investigated. Liu et al. [10] developed a frailty phenotype in aged C57BL/6 mice that matched the clinical criteria used by Fried et al. [4], and included grip strength, walking speed, physical activity, and endurance that were evaluated by the inverted-cling grip test, rotarod, voluntary wheel running, and an endurance score obtained from the grip test and rotarod. Similarly, Parks et al. [11] established a frailty index based on accumulated deficits derived from Rockwood and colleagues [5,6], in which aging C57BL/6 mice were assessed using 31 items that included both invasive and noninvasive variables (i.e., activity levels, hemodynamic measures, body composition and basic metabolic status). This group later developed a more simplified version of their 31-item frailty index that only used readily apparent noninvasive signs of clinical deterioration [12]. The development of animal models for frailty represents an important step forward in how we understand frailty, and interventions that may delay or prevent its progression [9].

Although these mouse models are integral to frailty research, they do have some limitations and can therefore be “fine-tuned” to further advance the field. For instance, the original [11] and modified [12] frailty indexes do not include important functional assessments such as walking speed and endurance; physiological variables known to decrease in aging rodents and humans [13,14]. The frailty phenotype established by Liu et al. [10] attempted to match the clinical criteria used in Fried’s human model [4], but did not include a weight factor, lacked an established endurance test and only assessed a small cohort of mice at a single age. However, probably the most important factor missing from these published frailty studies is the use of a phenotypic approach with a longitudinal component that explores important characteristics of frailty, including the onset and prevalence of frailty, and overall healthspan and mortality.

Therefore, the overall goals of this study were first to identify the onset and the prevalence of frailty across the lifespan of C57BL/6 male mice. Second, to determine if a frailty phenotype predicts mortality. To accomplish these goals, we assessed body weight, body fat percentage, walking speed, strength, endurance and physical activity in male C57BL/6 mice longitudinally, in which the same cohort of mice were tested across their entire lifespan.

## RESULTS

### Mouse mortality

**Figure 1:**
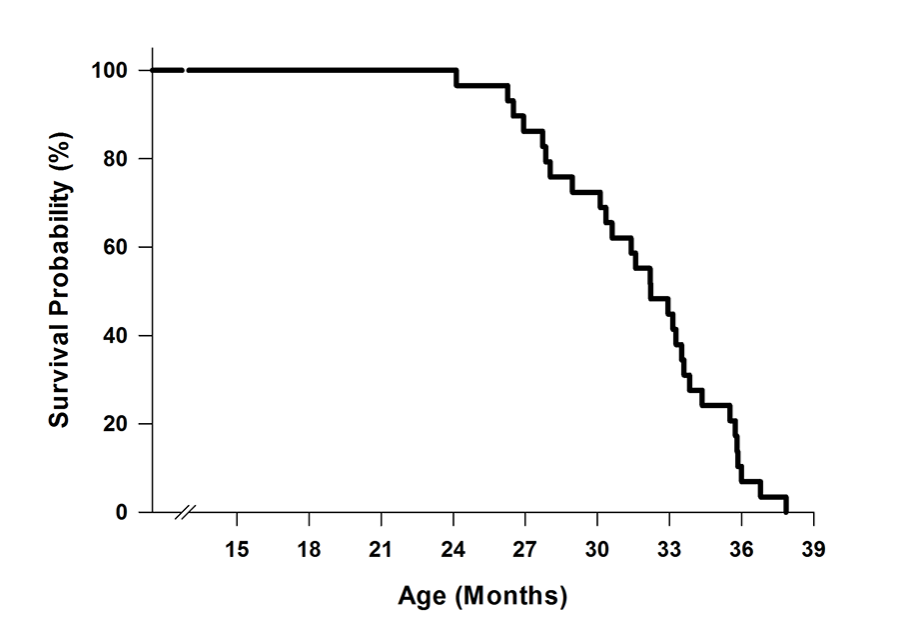
**Survival curve of C57BL/6 mice (n=29).** Mice were aged in the Animal Science Center at Boston University. Mortality occurred when mice either died unexpectedly or were euthanized due to morbidity.

Figure 1 shows the survival curve of 29 male mice. The mean survival age was 31.83±0.67 months, with the first and last mouse dying at 24.13 and 37.84 months, respectively.

### Identifying frail mice

**Figure 2:**
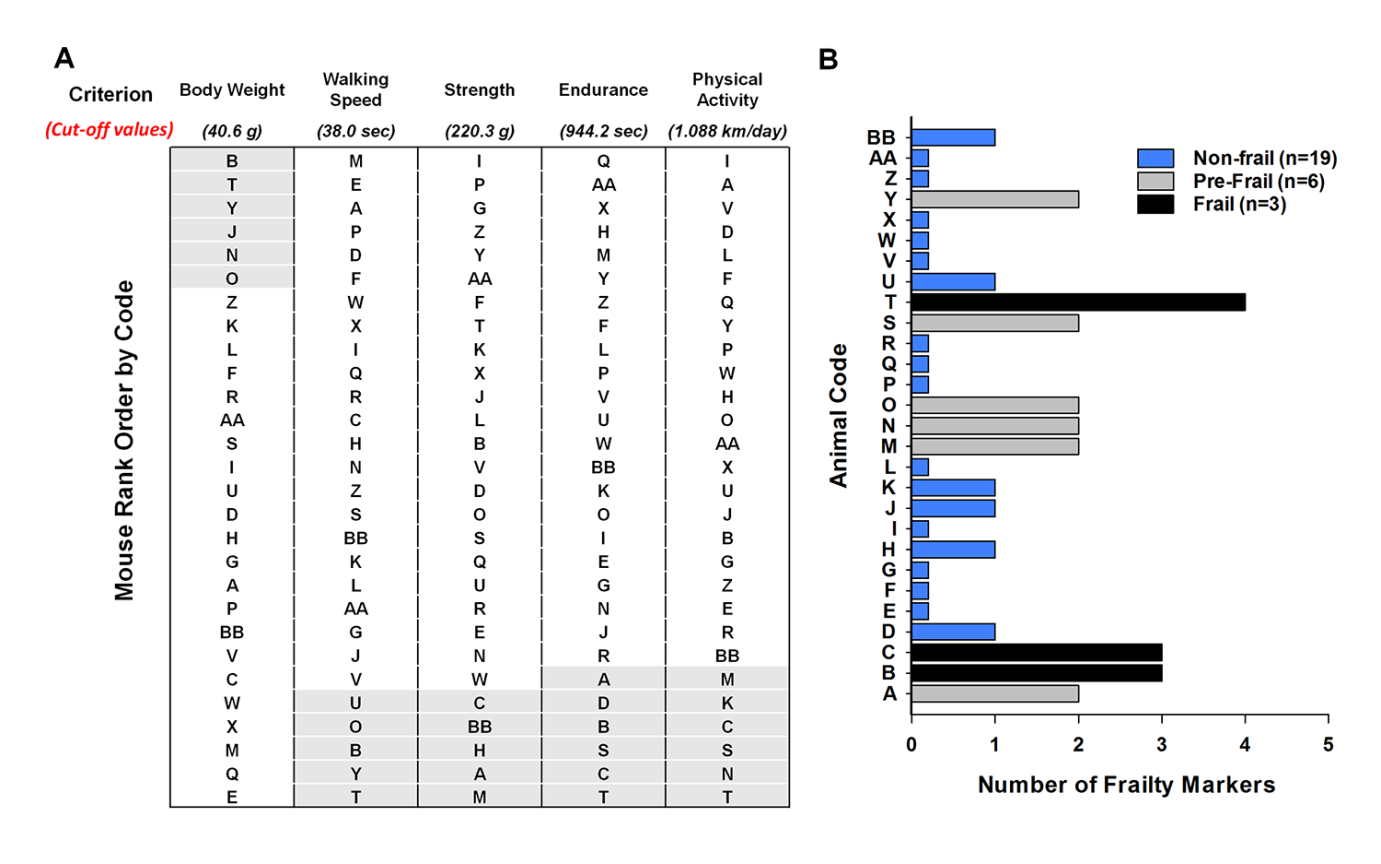
**Frailty status of mice at 23 months of age.** (**A**) The mice were coded A-Z, AA, and BB and rank – ordered by performance with the exception of body weight. For body weight the mice were ranked from heaviest to lightest. The cut-off values of each criterion (body weight, walking speed, strength, endurance and physical activity) are shown in parentheses. The shaded areas (light grey) identify the mice in the bottom 20% for performance and the top 20% for body weight. (**B**) Number of frailty markers for each mouse at 23 months of age. Frailty was defined if the mouse presented with three or more of the criterion markers (below or above the cut-off points); whereas, pre-frailty was designated if the mouse presented with two frailty markers. Mouse B, C, and T were identified as frail (black). Mouse A, M, N, O, S, and Y were identified as pre-frail (grey). The remaining mice were identified as non-frail (blue).

The cut-off values for each frailty criterion (20^th^ percentile) and the mouse rank order for each criterion from best to worst with the exception of body weight are listed in Figure 2A. Mice were identified to have a positive marker of frailty if the mice performed below the cut-off value for walking speed (38.0 sec), strength (220.3 g), endurance (944.2 sec), and physical activity (1.088 km/day). For body weight, the mice were positive for the frailty marker if they weighed 40.6 g or greater. Using this approach, we identified 19 mice as non-frail, 6 mice as pre-frail, and 3 mice as frail at 23 months of age (Figure 2B).

### Onset and prevalence of frailty

**Figure 3:**
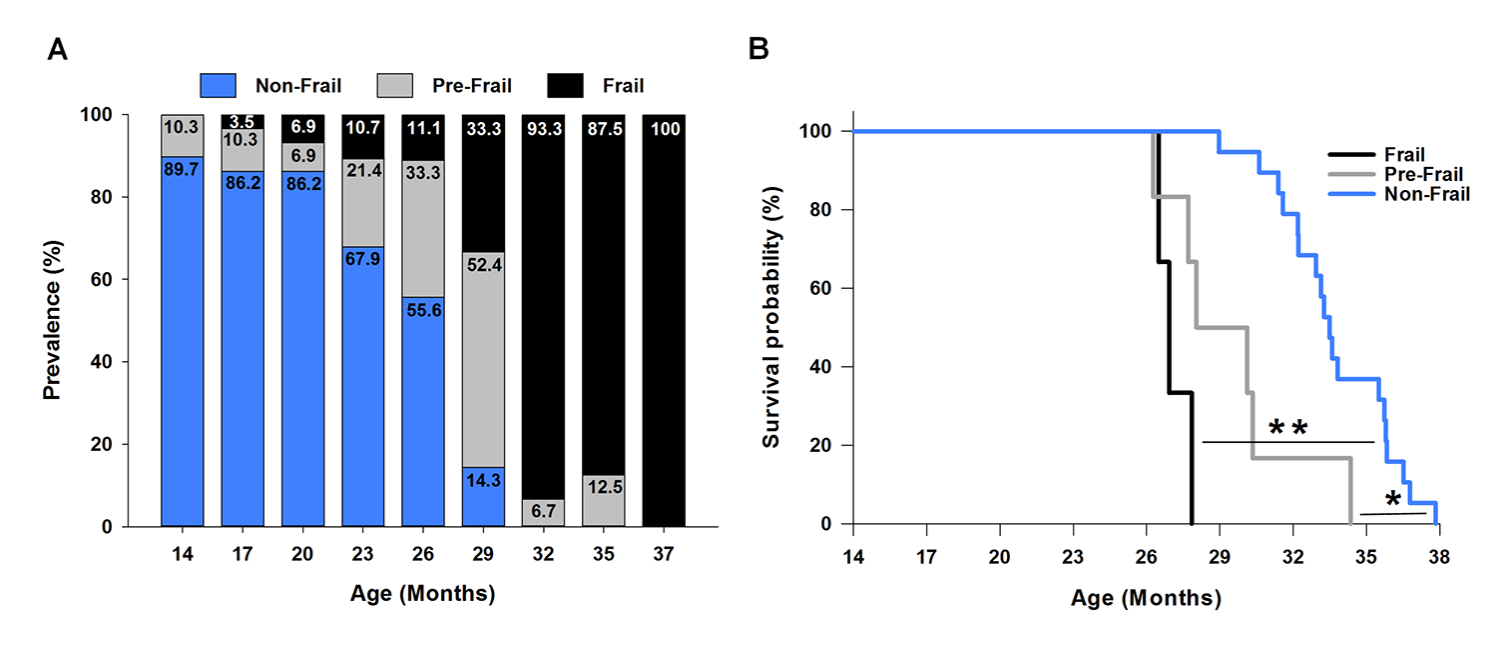
**Frailty: onset, prevalence and mortality.** (**A**) The prevalence of frailty across the lifespan. The frailty status was based on the cut-off values of each criterion determined at 23 months of age (Figure 2). The numbers within each bar graph represent the percentage associated with frail (black), pre-frail (grey), and non-frail (blue) for each age. The onset of frailty was identified at 17 months of age (3.5% prevalence). (**B**) Kaplan-Meier survival curves estimated over the lifespan by frailty status. * indicates p<0.05 comparing pre-frail (grey curve) to non-frail (blue curve). ** indicates p<0.001 comparing frail (black curve) to non-frail (blue curve).

Next, using the cut-off values calculated at 23 months (Table 1, Figure 2A), the onset and prevalence of frailty were determined for all other age brackets (Figure 3A). At 14 months of age 10.3% of the mice were pre-frail, and by 29 months increased and peaked at 52.4%. Following 29 months this percentage declined, with 6.7 and 12.5% of mice being identified as pre-fail at 32 and 35 months, respectively. The onset of frailty occurred at 17 months, and represented only 3.5% of the mouse cohort (1/29 mice). The percentage of frail mice steadily increased up to 33.3% at 29 months. Beyond 29 months, basically every remaining mouse was identified as frail (i.e., 87.5-100%) (Figure 3A).

**Table 1:** Frailty criteria and cut-off values.

**Human Frailty Phenotype** **Fried et al., 2001**	**Mouse Frailty Phenotype** **New Approach**	**Cut-off Values**
Low Activity	Voluntary Wheel Running	Lower 20% (1.088 km/day)
Poor Endurance	Treadmill Test	Lower 20% (944.2 sec)
Weakness	Grip Meter	Lower 20% (220.3 g)
Slowness	Rotarod Test	Lower 20% (38.0 sec)
Unintentional Weight Loss	Body Weight	Upper 20% (40.6 g)

### Predicting mortality of frail mice

To determine if our frailty criteria could accurately predict mortality, mice identified as frail, pre-frail and non-frail at 23 months (Figure 2B) were assessed using a Kaplan-Meier survival analysis (Figure 3B). The three frail mice had a mean survival time of 27.09±0.40 months and 100% probability of dying before 28 months. Five of the six pre-frail mice died before 31 month (83% probability) and had a mean survival time of 29.48±1.16 months. The mean survival time of the non-frail mice (n=19) was 33.75±0.54 months. Analyzing the three groups, non-frail mice lived longer than pre-frail and frail mice (p=0.004 and p<0.001), and pre-frail mice tended to live longer than frail mice (p=0.09).

### Comparison between frail/pre-frail and non-frail mice early in life

**Figure 4:**
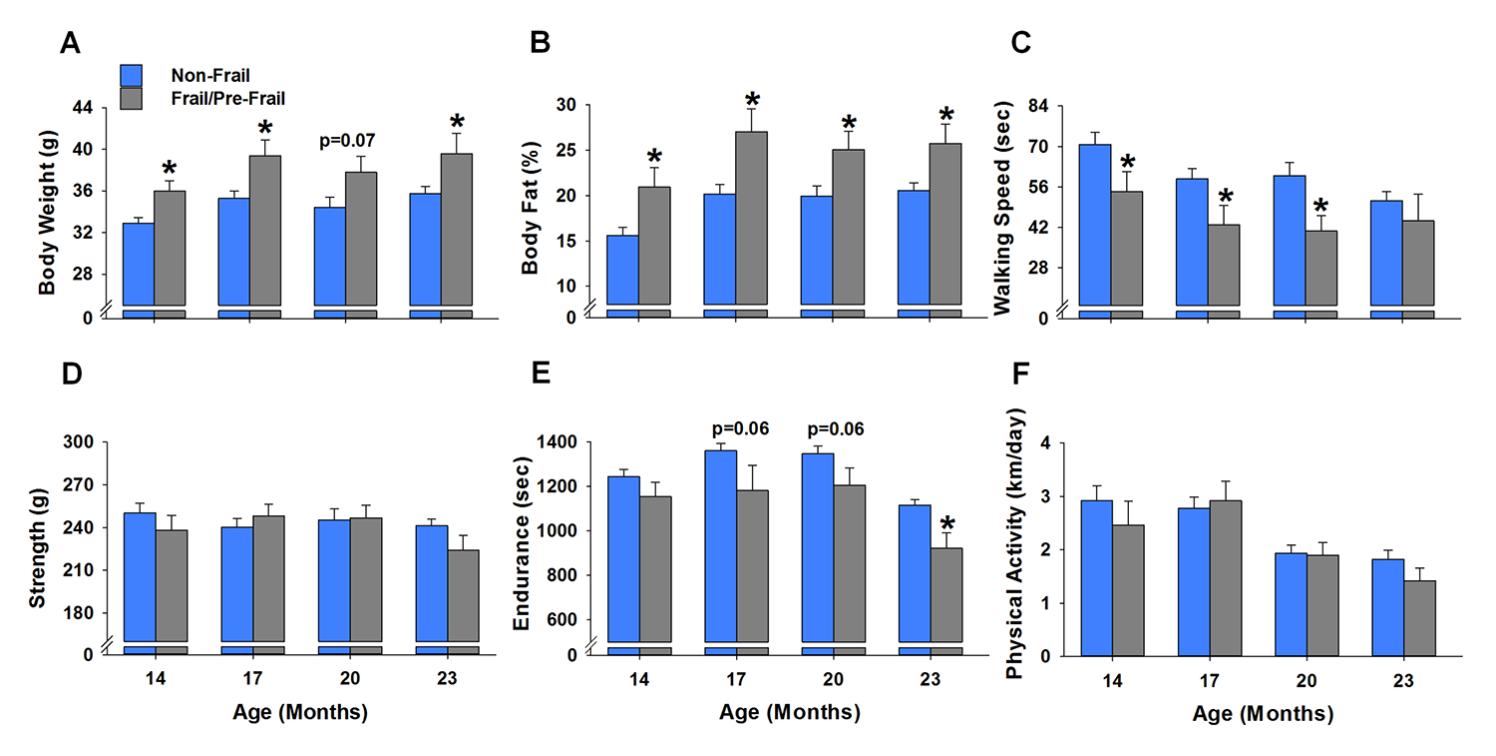
**Early age differences between frail/pre-frail and non-frail mice.** Mice were identified as frail/pre-frail or non-frail based on the cut-off values of each criterion determined at 23 months of age. (**A-F**) summarize the performance differences in these mice at ages of 14, 17, 20 and 23 months. Values are presented as mean ± SEM. * indicates p<0.05 comparing frail/pre-frail to non-frail.

To assess if the mice identified as frail or pre-frail at 23 months of age (i.e., mice A, B, C, M, N, O, S, T, Y) presented morphological and/or functional differences from the non-frail mice, the frail and pre-frail mice were grouped together and compared to the non-frail mice (Figure 4A-4F). Frail/pre-frail mice weighed 10-12% more and possessed 25-34% more body fat than the non-frail mice between 14 and 23 months of age (p≤0.03), besides for body weight at 20 months (p=0.07) (Figure 4A & 4B). As with body weight and body fat percentage, walking speed was different between frail/pre-frail and non-frail mice earlier in life, with time spent on the rotarod being 23-32% less at 14, 17 and 20 months (p≤0.046) (Figure 4C). Endurance measured as treadmill time to fatigue was 17% less in frail/pre-frail mice when compared to non-frail mice at 23 months (p=0.003) (Figure 4E). There were no differences in grip strength or physical activity between frail/pre-frail and non-frail mice between 14 and 23 months of age (p≥0.09) (Figure 4D & 4F).

### Age-related changes

Because of the longitudinal research design used in this study, it was possible to evaluate age-related changes in the same mice across several testing periods. For this analysis a subset of 15 mice from 14 to 32 months of age was assessed. In this way, a repeated measures ANOVA could be used across seven testing periods in mice that died at ~32 months, the mean survival time of the group.

### Body weight and body fat percentage

**Figure 5:**
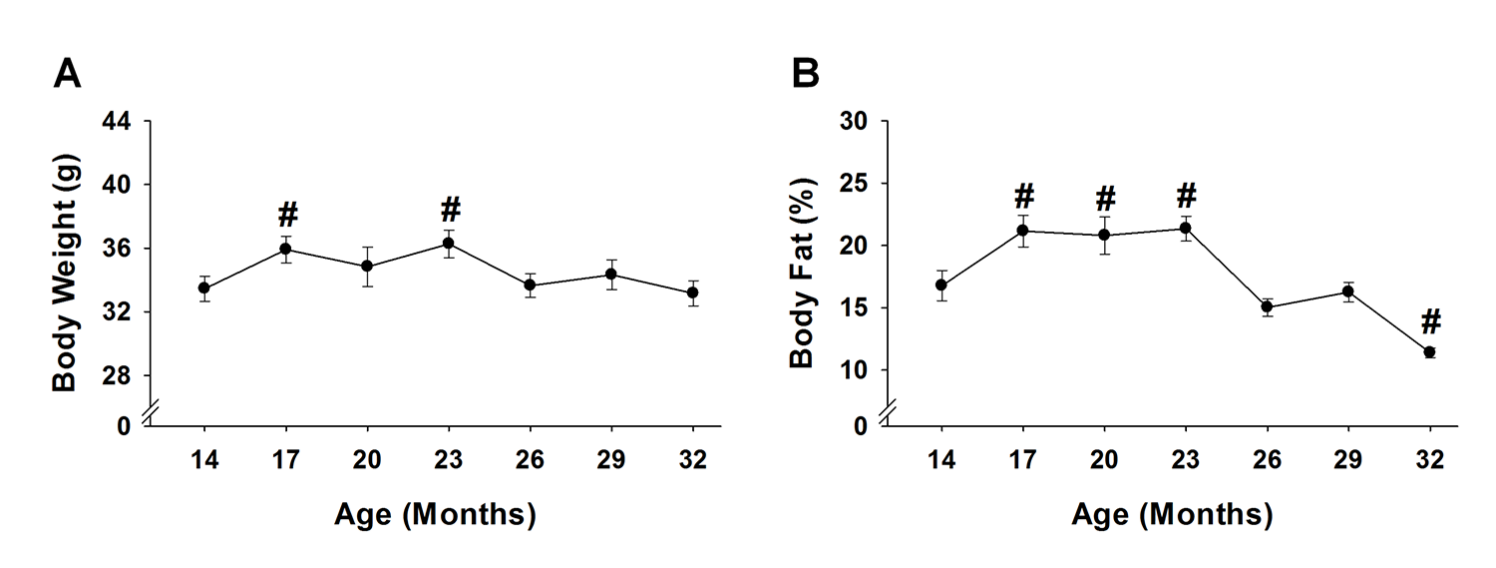
**Body weight and body fat % across the lifespan.** Body weight (**A**) and body fat % (**B**) of 15 mice were analyzed to test age-related changes using one-way repeated measures ANOVA followed by Bonferroni post-hoc. #, p<0.05 compared to 14 months of age.

**Figure 6:**
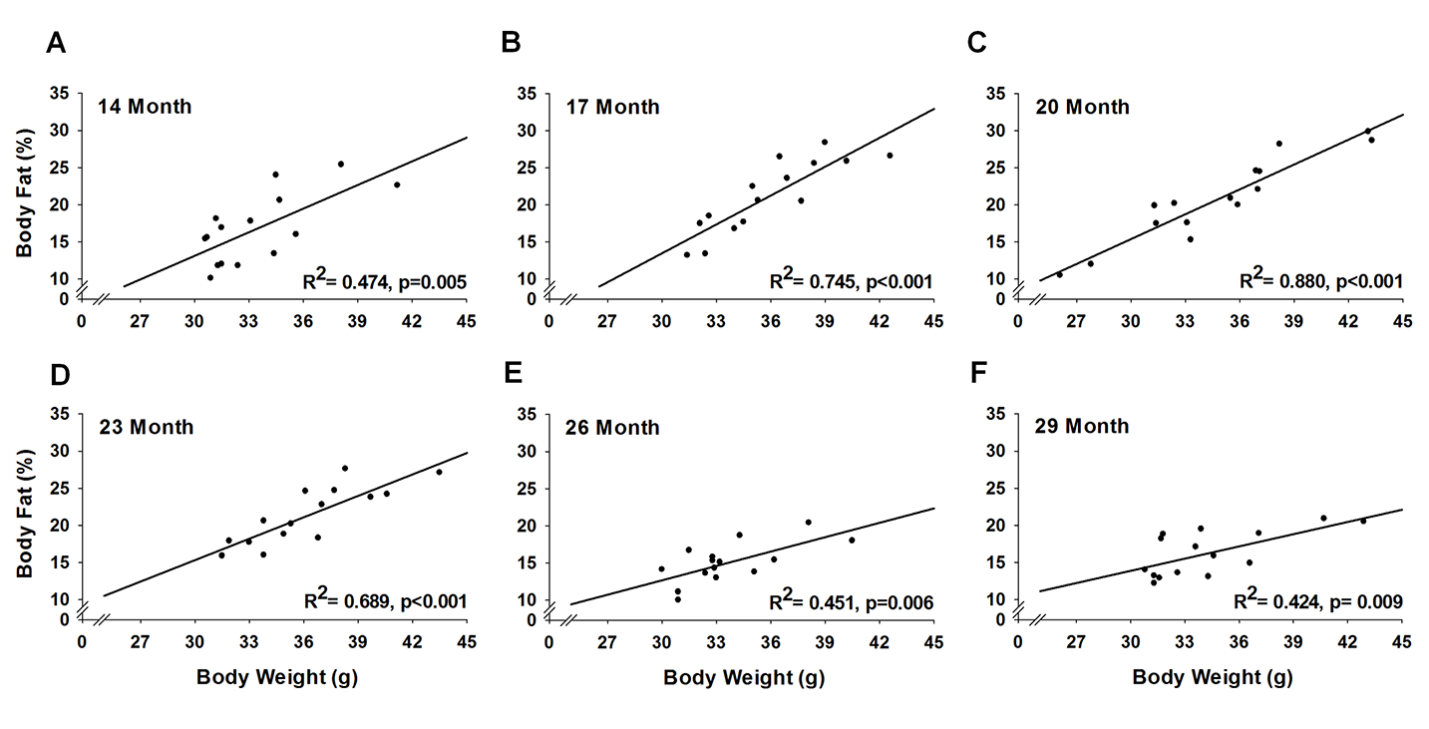
**Relationship of body weight and body fat % across the lifespan.** For each testing period, 15 mice were analyzed to test the relationship between body weight and body fat % using a simple linear regression.

Body weight increased 7-8% at 17 and 23 months (p≤0.02), and returned to baseline at 26 months and remained stable until 32 months (Figure 5A). As with body weight, body fat percentage also increased, but to a greater extent. From 17 to 23 months, body fat percentage increased 24-27% (p≤0.03) before decreasing 32% (p=0.03) at 32 months (Figure 5B). Regression analysis revealed that body weight and body fat percentage were related from 14 to 29 months (Figure 6A-6F), reaching a peak correlation of 0.88 (p<0.001) at 20 months (Figure 6C). No relationship was observed at 32 months (R^2^=0.05, p=0.433, data not shown).

### Functional characteristics

**Figure 7:**
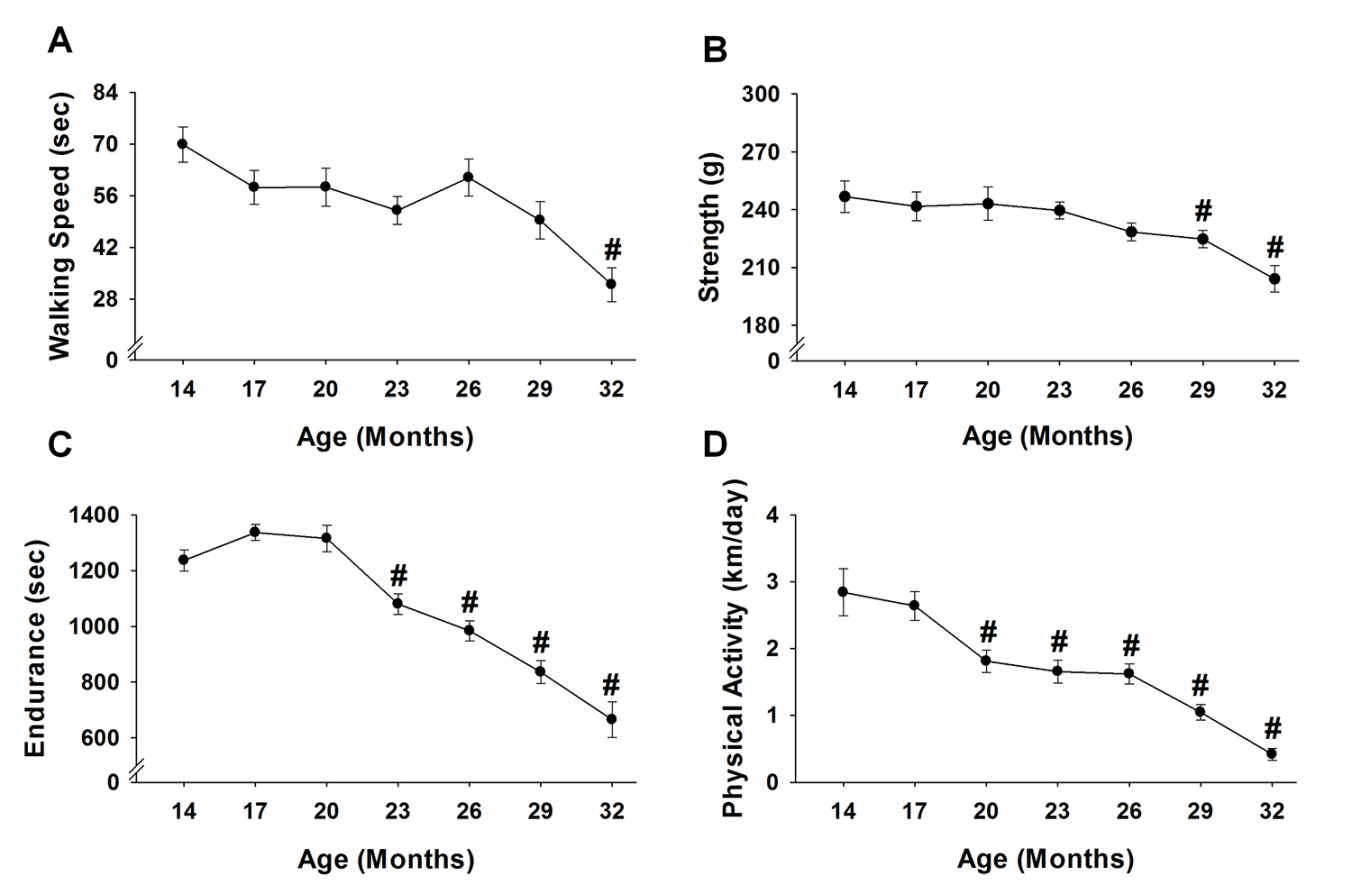
**Performance across the lifespan.** Fifteen mice were analyzed to test age-related changes using one-way repeated measures ANOVA followed by Bonferroni post-hoc for walking speed (**A**), strength (**B**), endurance (**C**), and physical activity (**D**). Values are presented as mean ± SEM. # significantly different from 14 months of age (p<0.05).

When compared to the initial baseline assessments performed at 14 months, age-related changes were observed for all functional characteristics (Figure 7A-7D). Physical activity determined by voluntary wheel running was the first variable to experience an age-related change, decreasing 55-85% from 20 to 32 months (p≤0.03) (Figure 7D). Endurance was the next variable to decrease, with mice time to fatigue declining 13-46% from 23 to 32 months (p≤0.046) (Figure 7C). Strength measured by a grip test decreased 9-17% from 29 to 32 months (p≤0.04) (Figure 7B). Walking speed as assessed by time on the rotarod was the last variable to experience an age-related change, decreasing 54% at 32 months (p<0.001) (Figure 7A).

## DISCUSSION

Frailty is a clinical syndrome defined as a late-life vulnerability to adverse health outcomes that ultimately leads to death. The development of animal models for frailty represents an important step forward in how we understand and treat frailty. Here, we expanded on a published mouse frailty phenotype [10] that was adopted from Fried et al. [4] that utilizes a multifaceted approach, incorporating body weight, weakness, poor endurance, slowness and low physical activity. However, our phenotype differed in that we selected a high body weight, rather than unintentional weight loss as a positive marker of frailty. Using our frailty phenotype in a longitudinal study design, we were able to define the onset and prevalence of frailty, and accurately predict mortality. We propose frailty phenotypes, such as the one presented here, are minimal effort, cost effective tools that can be used to determine biomarkers, mechanisms and potential treatments for frailty.

Using a mouse frailty phenotype focused on physiological function it is possible to identify two characteristics of frailty, the onset and the prevalence of frailty across the lifespan. In the present study, the onset of frailty occurred at 17 months when there was a 100% survival rate of our cohort. The prevalence of frailty increased across the lifespan, with nearly every mouse being identified as frail by 32 months (52% survival rate) (Figure 3A). These ages in mice represent human years of ~60 and over [13,16]. Our data are not consistent with a recent report, demonstrating no change in the prevalence of frailty across the lifespan in female ICR/CD1 mice when using the Valencia Score frailty phenotype [17]. Yet, the overall prevalence of frailty increases as humans age, with 5-10% in 60-69 years old to 26-65% in those aged ≥85 years of age [1,7,8]. The increasing prevalence of frailty in both humans and rodents with age suggests common etiologies underlying the frailty syndrome. Interestingly, the degree of prevalence in the very old is different between research performed in the laboratory with rodents and in clinically orientated human research. There could be many underlying reasons for this difference including factors specific to human frailty, such as socioeconomic status and the race/ethnicity of the population. Because these factors can be controlled in rodent studies the assessment of frailty in preclinical models becomes very important. In contrast to the reported prevalence of frailty, to our knowledge the age at which frailty is first observed has not yet been reported in male C57BL/6 mice. The value of identifying the onset of frailty is important in teasing out the underlying etiologies of frailty; however, the identification of this critical time point requires a longitudinal research design, which at times, is prohibitive.

As expected, we report a strong association between mouse frailty status at 23 months of age and overall mortality. Using the five criteria (Table 1), we were able to accurately predict that frail and pre-fail mice die before non-frail mice (Figure 3B). In fact, every frail mouse died within five months (28 months of age), while the majority of the pre-frail mice died in eight months (31 months of age). These finding are evident when comparing the lifespan of frail, pre-frail and non-frail mice; non-frail mice (33.8 months) lived longer than mice identified as pre-frail (29.5 months) and frail (27.1 months). Similar associations have been reported using an accumulation deficit frailty assessment in mice, rats and humans [18–20], and using the Valencia Score in female ICR/CD1 mice [17]. Overall, these findings emphasize the negative outcomes of the frailty status across species.

Early identification of individuals at risk of developing frailty, even at a pre-frail status, has clinical, social and economic benefits due to the burden this syndrome poses on society. For instance, from a quality of life perspective there is evidence attesting that frailty and pre-frailty can be prevented or delayed, by the incorporation of timely and appropriate interventions [21]. The frailty phenotype utilized in the present study focused largely on physiological function, thereby incorporating several tissue/organ systems (e.g., neuromuscular, cardiovascular, pulmonary). It is possible the cellular and molecular processes that maintain these systems were compromised and progressively deteriorated in the frail/pre-frail mice. The increased prevalence of frailty with aging likely represents decreased resilience to ongoing stress, which ultimately could lead to extensive cellular alterations. Some of these alterations include genomic instability, mutations, altered gene expression, loss of cell division potential, cell death and/or impaired intercellular communication [22]. Hence, identifying the onset and prevalence of frailty across the lifespan, in addition to predicting mortality, has the potential to yield information about susceptibility and resilience to the aging processes. Further investigations will be needed to determine which cellular and molecular processes are compromised in frail animals/humans, and if attenuating these compromised processes influence healthspan and/or mortality.

An important and novel component of our frailty model is the criterion of a high body weight (Table 1), which does not follow unintentional weight loss outlined by Fried et al. [4]. Others have applied similar weight loss criterion to mouse models of frailty, for instance Gomez-Cabrera et al. [23] considered mice that lost more than 5% of their weight at 17 months of age to have a positive marker of frailty. However, we would like to emphasize three key concepts. First, our data do indeed support that in a group of healthy mice, body weight steadily decreases from 23 to 32 months of age (p<0.01, Figure 5A). Second, by the mean survival age of our mouse cohort (i.e., 32 months), many of the mice that died before this point were actually the heaviest mice earlier in life (Figure 4A). Lastly, even though the heavy mice died early, it does not necessarily imply these mice are impervious to the weight loss criterion established in human [4] and mouse [23] frailty phenotypes. Rather, it is possible the heavy mice that were identified as frail in this study lost weight soon before dying (i.e., days to weeks), but it was not captured due to the time between assessments. We would also add that it would be advantageous to implement interventions before a significant amount of weight is lost, as death would likely be inevitable at that point.

From this, we determined that in order to accurately predict frailty, one must consider body weight of the mice at all ages. Mice that weighed in the top 20% of our cohort at 23 months were therefore considered to have a positive marker of frailty. Although we are the first to suggest that high body weight be considered a criterion to predict mouse frailty, others have suggested body weight negatively impacts longevity across several mammalian species [24]. In mice, the most basic examples include the Ames dwarf that has superior longevity [25,26] and calorie restriction, in which reducing body weight increases the mean and maximum lifespan [27,28]. Moreover, in a series of 15 mouse stocks that were selected over 22 generations for their different rate of body weight, body weights at 3, 6 and 12 months were significant predictors of longevity (among stocks) [29]. Most relevant to the present study was work done by Miller et al. [30], who reported a significant association between body weight and mortality in mice 2 to 24 months of age. In line with this, our data suggest that frail/pre-frail mice are heavier than non-frail mice throughout adulthood (Figure 4A). Collectively, these data strongly suggest that high body weight early in life negatively impacts healthspan and longevity.

Mice with high body weights also possessed the most body fat (Figure 6A-6F), indicating the “extra weight” was not coming from increased muscle mass. Indeed, the relationship between body weight and body fat has been observed across various species, including humans [31–33]. Furthermore, epidemiological observations provide solid evidence that mortality is associated with excessive body fat (i.e., obesity) [34–36]. Miller et al. [30] suggested that some of the genetic variations that control the speed of body growth in young mammals also lead to alterations in the stress resistance of many cell types. Moreover, these changes in cellular stress resistance endure through most or all of the lifespan, thus influencing the timing and severity of multiple forms of late‐life, potentially fatal, illnesses [30]. This hypothesis aligns with the concept frail mice lack resilience to ongoing stress, in that frail mice may exhibit deficits across various tissue/organ systems, which can be indirectly measured using a phenotypic approach. For instance, our data demonstrate frail/pre-frail mice were heavier, possessed more body fat and were less physically fit across most of their adult lives when compared to non-frail mice (Figure 4A-4C).

Using tests of physiological function it is possible to indirectly assess the health of many tissue/organ systems. Moreover, using our longitudinal study design, we were able to evaluate these systems in the same mouse over a lifetime. Age-related deficits were recorded in walking speed, strength, endurance, and physical activity (Figure 7A-7D), similar to that observed by others using cross-sectional research designs [14,23]. Importantly, our functional variables followed that of the physical phenotype established by Liu et al. [10], with the addition of an established endurance test, time to fatigue on a motorized treadmill. The frailty phenotype presented here is unique when compared to deficits models that mainly use clinical signs of frailty (e.g., fur color, hearing loss, vision loss, loss of whiskers, etc.) [11,12]. In that, our phenotypic model is objective, it functionally challenges the mouse (i.e., maximal treadmill running) and depicts that frail/pre-frail are dysfunctional early in life. In fact, mice identified as frail/pre-frail at 23 months were typically slower and less aerobically fit earlier in life when compared to non-frail mice (Figure 4C & 4E). Taken together, a clear relationship exists with frail/pre-frail mice being unhealthy early in life, as assessed by body weight, obesity and physiological function.

Although our frailty phenotype accurately predicts mortality, future studies will be needed to extend upon these findings. For instance, C57BL/6 male mice were utilized, and although this strain is widely used, other mouse strains may need to be tested for frailty at different time points, depending on their individual lifespans. Moreover, studies will need to assess if frailty in female mice is similar to males, due to declining levels of estrogen that occur with ovarian senescence. Despite continued research, we believe reduced physical function and obesity early in life could be used as “universal” frailty criteria for all strains and across genders.

The frailty phenotype utilized in the present study focused largely on body weight and physiological function, thereby incorporating several tissue/organ systems. Our data demonstrate frail mice were obese and physically unfit throughout the majority of their adult lives, and had a reduced lifespan. Using a frailty phenotypic approach, we were able to identify that the onset of frailty begins the second half of life and steadily increases with age. We suggest that frail mice lack resilience to the aging process, in that multiple tissue/organ systems deteriorate at an accelerated rate compared to non-frail, healthy mice, which ultimately leads to early mortality. In closing, identifying the onset and prevalence of frailty across the lifespan, in addition to predicting mortality, has potential to yield information about susceptibility and resilience to the aging processes.

## MATERIALS AND METHODS

### Ethical approval and animals

Thirty-two C57BL/6 male mice were purchased from Jackson Laboratory (Bar Harbor, ME, USA) at 13 months of age. Mice were housed under a 12 hour light:dark cycle at 20-23^o^C in specific pathogen-free facilities and supplied with food and water *ad libitum*. Mice were allowed to age and die of natural causes. In the event that a mouse had to be euthanized due to factors outside of normal aging, as determined by the veterinary staff, the mouse was euthanized by inhalation of CO_2_. From the initial cohort of 32 mice, 3 mice were euthanized between 16 and 17 months of age due to non-age-related causes (e.g., over-grooming), leaving 29 total mice for this study. All animal procedures were in accordance with the standards set by the Institutional Animal Care and Use Committees at the University of Minnesota and Boston University.

### Experimental design

**Figure 8:**
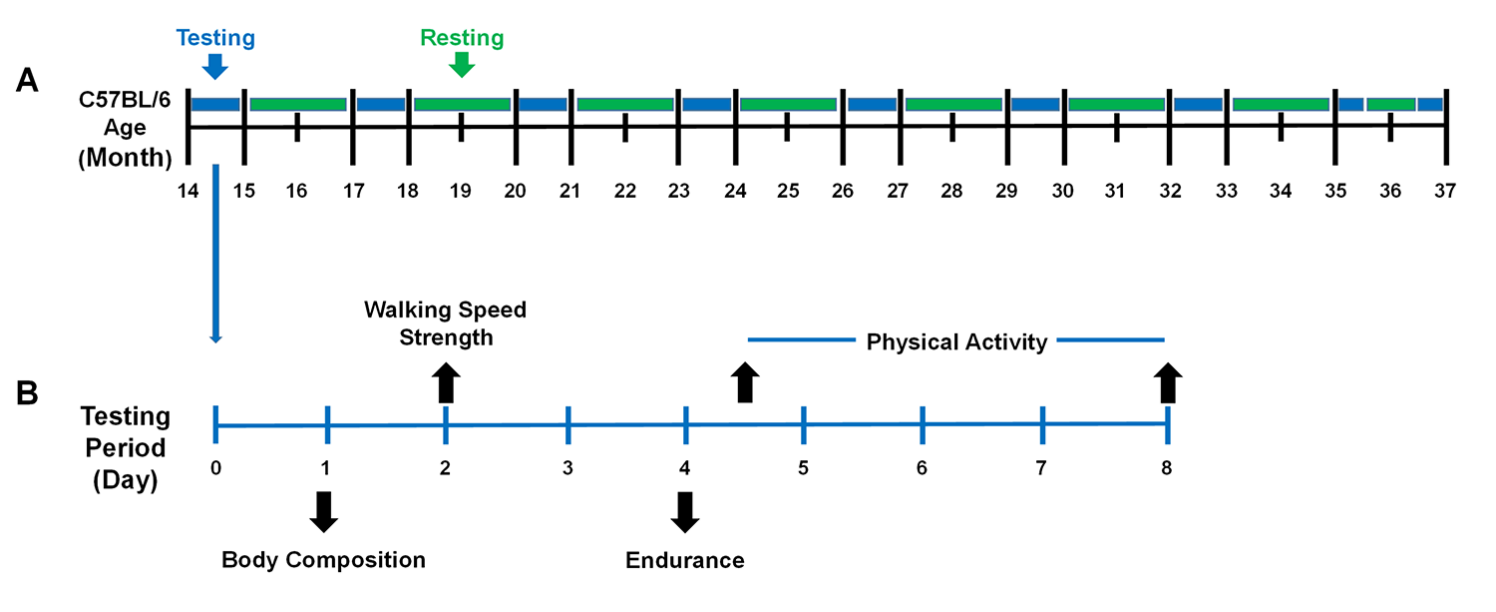
**The performance testing timeline of the mice across the lifespan.** (**A**) Mice were evaluated periodically between 14 – 37 months of age. Mice were housed in their cages without testing (green) and were evaluated for performance (blue) every three months. It required one month to test the entire cohort (blue) because 8 mice were evaluated each week during the testing period. (**B**) During the testing period, mice were evaluated for frailty using the five criteria (body composition, walking speed, strength, endurance, and physical activity). Day 1: Body composition included body weight and body fat percentage assessed by Dual-energy X-ray absorptiometry (DEXA). Day 2: Walking speed and strength were evaluated by rotarod and a grip strength meter. Day 4: Endurance was evaluated using a treadmill test and then the mice were placed in voluntary wheel running cages. Day 8: Mice were returned to their original cages. A total of 4 testing periods were performed in a month.

Performance testing was initiated at 14 months of age, and continued every 3 months (17, 20, 23,… 36 or death). Importantly, this repeated measures research design allowed us to track each individual mouse across its lifespan and determine when it died. For each performance testing period, mice were subjected to a battery of assessments over a one-week period during every three-month interval (Figure 8A & 8B). All testing procedures followed the same protocol during each assessment, and to ensure testing reliability, all assessments were completed by the same testers.

### Body weight and body fat percentage

All mice were weighed on an electronic scale (Portable scales: CS-200, Ohaus, Parsippany, NJ, USA). Once body weight was obtained, body fat percentage was evaluated using a Lunar PIXImus densitometer (GE Lunar Corporation, Madison, WI, USA) in which mice were anesthetized with isoflurane, carefully placed on an adhesive specimen tray and scanned. Measurements were obtained with the skull excluded and tails included, to increase accuracy as recommended by the manufacturer. Quality control included a phantom mouse as a calibration standard that was done prior to each testing day.

### Walking speed

Walking speed was evaluated using a rotarod (Rota-Rod R/S; LSi Letica, Cornella, Spain). As a warm-up, mice were placed on the rotarod and walked at 4 rpm for 30 seconds. Following the warm-up, rotarod speed increased 1 rpm every 8 seconds up to 40 rpm over a 5 min period. Walking speed was recorded when the mouse was unable to sustain the rotation speed of the rotarod. Each mouse performed three trials with a 10-minute rest period in-between each trial. The best score of these trials, recorded as seconds, was used as walking speed.

### Strength

Strength was evaluated using a grip meter test (Grip meter; P/N760483, Coulbourn Instruments, Whitehall PA). Mice were gently lowered over the top of a wire grid so that the front and hind paws gripped the grid. Once gripped, the tail of each mouse was pulled back steadily, keeping the mouse’s torso in a horizontal position. When the mouse was unable to maintain its grip, the trial was over and the grip strength, in grams, was recorded. Each mouse performed two trials with a 10-minute rest period in-between each trial. The best score of these trials was used as peak grip strength.

### Endurance

Endurance was evaluated using a time to fatigue test on a motorized treadmill (Exer 3/6 Treadmill; Columbus Instruments, Columbus, OH). The protocol started with a brief warm-up at 5 m/min for 5 min. After the warm-up, the mice remained on the treadmill and the time to fatigue test began, in which a ramp protocol was used with speed increasing 1 m/min every minute. Exercise motivation was provided by gently tapping the mouse’s rear, as previously described [15]. Time to fatigue was recorded following the third time the mouse could no longer keep pace with the speed of the treadmill (e.g., the mouse remained at the back of the treadmill for three seconds without attempting to re-engage). Endurance was determined to be the total amount of time, in seconds, the mouse remained on the treadmill.

### Physical activity

Physical activity was evaluated by assessing voluntary distance ran using a running wheel (Model number: 80820F, Lafayette Instruments, Lafayette, IN). Briefly, mice were individually housed in the wheel running cages for four days. The running distance, in revolutions, was recorded and converted to kilometer. The average distance ran per day was used to score physical activity.

### Frailty criteria

A frailty phenotype was modified from that of Liu et al. [10] who selected physical assessments based on the human clinical criteria described by Fried et al. [4] (Table 1). Thus, our frailty phenotype included the following physical components: body weight, walking speed (rotarod speed), strength (grip strength), endurance (time to fatigue) and physical activity (voluntary wheel running distance).

Following the percentiles used by Fried et al. [4], mice that fell in the bottom 20% of our cohort for rotarod speed, grip strength, time to fatigue or voluntary wheel running distance were considered positive for frailty (i.e., for that given criterion). However, rather than unintentional weight loss, we determined that mice with a high body weight, in which they weighed in the top 20% to be positive for this frailty criterion (i.e., body weight). A detailed rationale for selecting heavy or overweight mice as a positive marker for frailty is provided in the discussion.

These criteria were used to identify frailty cut-off values at 23 months of age (Table 1). Mice with three or more positive frailty markers were identified as frail, mice with two positive markers were identified as pre-frail and mice with one or no positive frailty marker were considered non-frail. The age of 23 months was specifically selected for several reasons. First, in our initial cohort (n=32, data not shown) and that published by National Institute of Aging (NIA), this age represents >75% survival, meaning it is near the maximal age before mice begin dying, making it an optimal age to predict frailty. Indeed, from 23-29 months of age, approximately 30% of the mice in our cohort died. Second, 23 months for a mouse is equal to ~65-75 human years [13,16], which corresponds to the initial age brackets assessed by Fried et al. [4]. Lastly, because frailty is thought to be reversible, this age provides adequate time to implement possible life changing interventions.

To determine if the frailty criteria outlined above could accurately predict mortality, survival curves were constructed on mice identified as frail, pre-frail and non-frail at 23 months of age. The cut-off values obtained at 23 months (Table 1) were then used to quantify the onset and prevalence of frailty for all other age groups (i.e., 14 to 37 months).

### Statistical analysis

A one-way repeated measures ANOVA (mouse x time) was used to test age-related changes in body weight, body fat, walking speed, strength, endurance and physical activity. In the event of a significant ANOVA, a Bonferroni post-hoc test was performed. The relationships between body weight and body fat percentage were fit with a simple linear regression and the square of the correlation coefficient (R^2^) was calculated. Differences between frail/pre-frail and non-frail were analyzed with an independent t-test. A Kaplan-Meier test was used to assess lifespan characteristics and for comparison between groups (i.e., frail, pre-frail and non-frail). An α level of < 0.05 was used for all analyses. Values are presented as mean ± SEM. Statistical analyses were performed with Sigma Plot 14.0 (Systat Software Inc., Point Richmond, CA) or SPSS 24.0 (IBM Corp, Armonk, NY) software.
